# Applied Scholarship in LGBTQ Aging: Implications for Policy and Practice

**DOI:** 10.1093/geroni/igaa057.2339

**Published:** 2020-12-16

**Authors:** Austin Oswald, Vanessa Fabbre

## Abstract

Lesbian, gay, bisexual, transgender, and queer (LGBTQ) elders have shown considerable strength while aging in a society predicated on heteronormative and binary expectations for gender and sexuality. The life trajectories of LGBTQ older adults are shaped by discrimination and stigmatization, and the embodied resistance that comes with demanding their full participation and recognition in society. This symposium highlights the innovative scholarship of emerging scholars in the field of LGBTQ aging who are engaging in diverse substantive and methodological investigations. The first study takes a comparative cohort approach to explore differences in stressors and depressive symptomatology between younger and older sexual minorities, highlighting the significance of cohort effects among LGBTQ people. The second paper uses data from the Health and Retirement Study to examine anticipated nursing home placement needs between LGB and heterosexual adults with suggestions to better prepare aging service networks. The third describes the influence of state legislature mandating LGBTQ-sensitivity training by examining differences in provider baseline knowledge and attitudes toward LGBTQ older adults in two states, one mandating LGBTQ-sensitivity training and one not. The final paper highlights findings from a multi-methods study that explores how long-term care workers, managers, and administrators respond when staff, visitors, or residents challenge LGBTQ rights for religious and moral reasons. Although substantively and methodologically varied, these studies all demonstrate the importance of applied scholarship that builds knowledge in support of policies and practices that promote equity among LGBTQ individuals across the life course. Rainbow Research Group Interest Group Sponsored Symposium.

